# Treatment of aortic thrombosis with retrievable stent filter and thrombolysis: a case report

**DOI:** 10.1186/s12872-019-1037-z

**Published:** 2019-03-05

**Authors:** Yonghua Bi, Hongmei Chen, Wenguang Zhang, Jianzhuang Ren, Xinwei Han

**Affiliations:** 1 Department of Interventional Radiology, the First Affiliated Hospital of Zhengzhou University, No.1, East Jian She Road, Zhengzhou, 450052 China; 2 Department of Ultrasound, Zhengzhou Central Hospital Affiliated to Zhengzhou University, Zhengzhou, China

**Keywords:** Retrieval stent filter (RSF), Aortic thrombosis, Transjugular intrahepatic portosystemic stent shunt (TIPSS), Complication

## Abstract

**Background:**

The retrievable stent filter (RSF) has been previously used for the treatment of vena cava thrombosis. In this study, the RSF was implanted to treat aortic thrombosis and then withdrawn.

**Case presentation:**

A 47-years-old woman presented with severe abdominal pain and fever. Computed tomography showed massive mural thrombosis in the thoracic and abdominal aorta complicated by portal venous thrombosis. The RSF was implanted, a transjugular intrahepatic portosystemic stent shunt was established and a thrombolytic catheter was inserted for portal vein thrombolysis. The aortic thrombus was successfully compressed and fixed without thrombosis. After 15 days, abdominal pain had ceased, the abdominal aortic thrombus was mostly dissolved and the RSF was retrieved. Catheter angiography confirmed the recovery of portal vein thrombosis.

**Conclusions:**

The RSF was able to compress and fix aortic thrombus without the usual complications of stenting after removal.

## Background

A large aortic thrombus may detach and cause fatal consequences at any time, such as blocking the visceral artery or the lower extremity arteries. However, traditional treatments are problematic. The retrievable stent filter (RSF) was previously used for the treatment of vena cava thrombosis [1–4]. Here we describe the successful use of an RSF that was implanted and then withdrawn for the treatment of aortic thrombosis in our department. The successful implementation of this new technology has opened up a new option for the treatment of aortic thrombosis, especially for giant thrombus in patients with thrombophilia.

## Case presentation

**Fig. 1:**
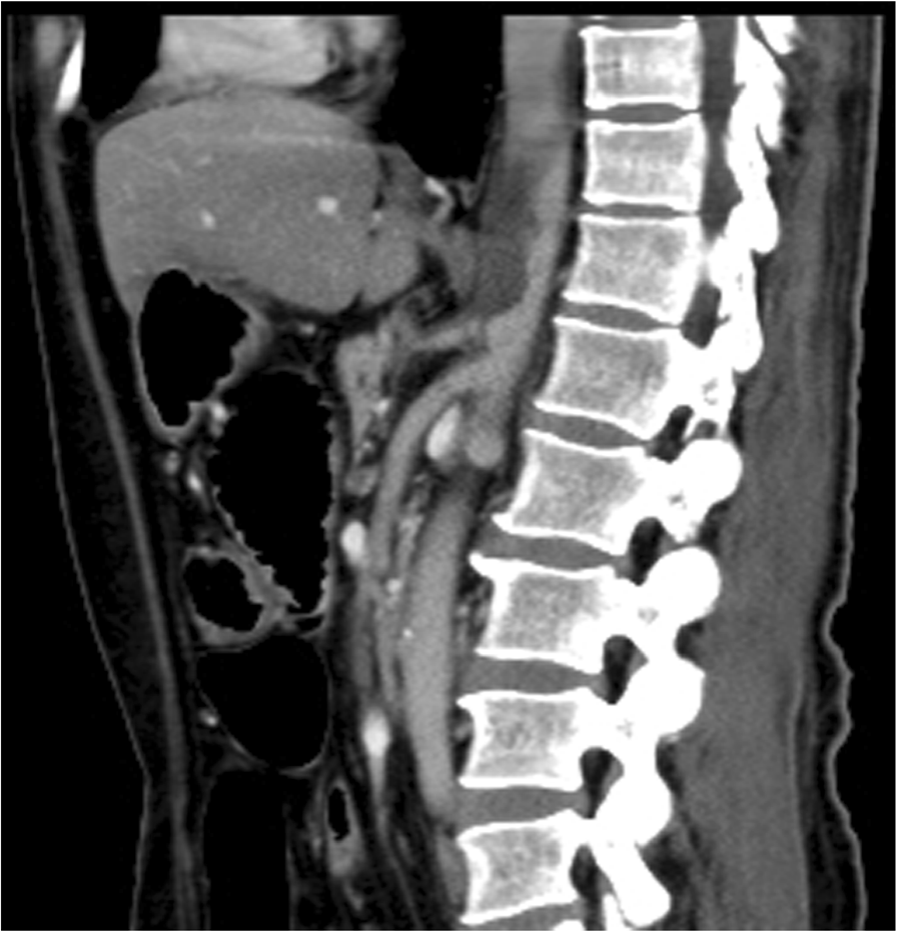
Computed tomography examination. Sagittal computed tomography scan showing aortic thrombosis

A 47-years-old woman with diabetes mellitus presented to our department with severe abdominal pain and fever. The local hospital’s computed tomography showed massive mural thrombosis in the thoracic and abdominal aorta from the level of the diaphragmatic muscle to the superior mesenteric artery (Fig. 1). The spleen had a large area of infarction complicated by portal venous thrombosis. This patient underwent amputation three years ago due to extensive thrombosis of the left upper extremity artery. Further examination in our hospital showed thrombosis in the portal vein, the superior mesenteric vein and the splenic vein. Laboratory examination showed the following: prothrombin time 10.9 s, D-Dimer 1.030 μg/mL, C-reactive protein > 200 mg/mL, erythrocyte sedimentation rate 99 mm/h, NH3 73.5 μmol/L. Rheumatic immune tests, liver function, kidney function and electrolytes were normal, except for an albumin of 25.6 g/L.

**Fig. 2:**
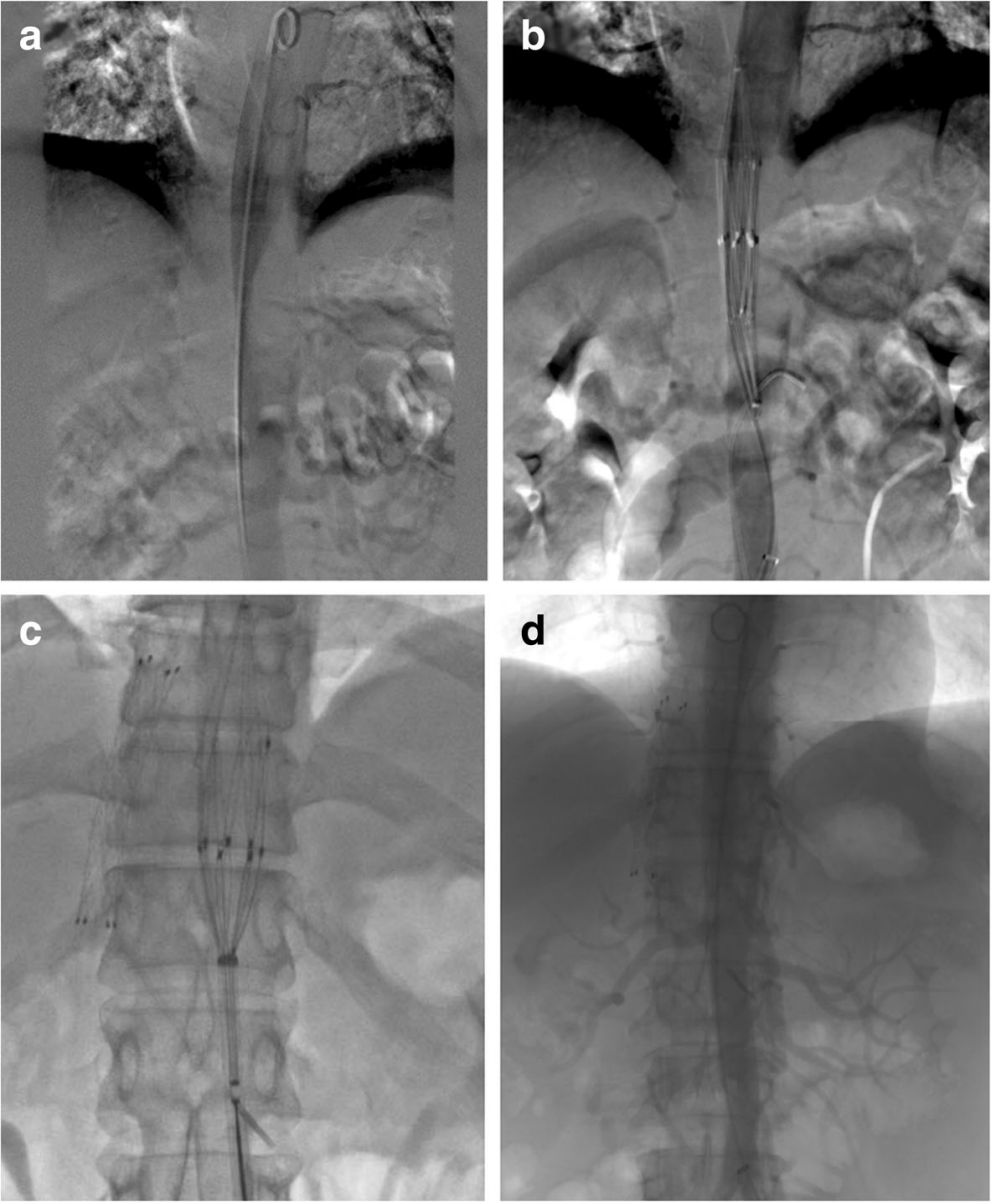
Aortic thrombosis was treated with the RSF and thrombolysis. Arrow showed thrombosis in aorta (**a**), the RSF was implanted in aorta **(b**), the RSF was retrieved after 15 days (**c**); aortic thrombus had almost disappeared (**d**)

**Fig. 3:**
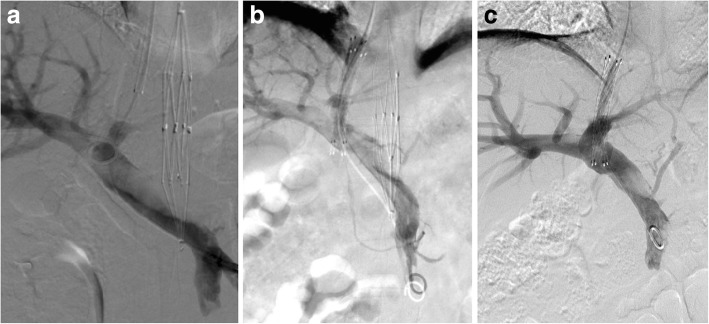
Portal vein thrombosis was treated with TIPSS and thrombolysis. Portal vein thrombosis was shown (**a**), TIPSS was conducted and a thrombolytic catheter was inserted for portal vein thrombolysis (**b**), little thrombus remained in portal vein after 15 days (**c**)

Preoperative preparation and intraoperative procedures were carefully performed to improve the success rate and to reduce the risk of thrombus shedding during intervention. The catheter and guide wire was placed in the mesenteric artery and left renal artery via left femoral artery puncture, so that balloon angioplasty or stent implantation could be performed immediately once those branch vessels were blocked by shedding thrombus. Written informed consent was obtained from the patient for the use of RFS and the right femoral artery was incised to implant the RSF. The aortic thrombus was successfully compressed and fixed without thrombosis during intervention (Fig. 2a, b). A transjugular intrahepatic portosystemic stent shunt (TIPSS) procedure was conducted and a thrombolytic catheter was inserted in the portal vein for thrombolysis (Fig. 3a, b). Urokinase 100,000 units (Lizhu pharmaceutical Co., Ltd., Guangdong, China) was dissolved in 50 ml of normal saline, and given by microinfusion pump every 8 h. Warfarin sodium tablets 3.75 mg (Qilu pharmaceutical, Shangdong, China) were taken orally once a day after the procedure. In addition, 1000 units of heparin sodium (Fengyuan pharmaceutical co., Ltd., Anhui, China) and 0.6 mg of octreotide (Chinese medicine & pharmaceutical co., Ltd., China) were dissolved in 50 ml of normal saline and given by microinfusion pump every 12 h. Omeprazole 40 mg (Changchun Fuchun Pharmaceutical Co., Ltd., Changchun, China) and levofloxacin 0.6 mg (Yangzijiang Pharmaceutical Group Co., Ltd., Jiangshu, China) were administrated intravenously once a day.

Relief of the patient’s abdominal pain was evident 3 days after the interventional procedure, and pain resolved completely after 15 days. Angiography showed that the abdominal aortic thrombus was mostly dissolved, with only a few residual thrombi. A 12 F sheath was introduced through the guide wire and the RSF was retrieved (Fig. 2c, d). Catheter angiography confirmed an recovery of portal vein thrombosis via indwelling catheter. Portal vein thrombosis almost completely disappeared and the patient was discharged after 16 days (Fig. 3c). She received oral warfarin sodium 100 mg per day for anticoagulation with an international normalized ratio of 2–3. One month later, the bilateral renal artery, superior mesenteric artery, lower extremity artery and portal vein were well visualized, without thrombosis. The upper abdominal aorta was normal with a small amount of residual thrombus. The patient appears normal and no complications have occurred after 14 months.

## Discussion

A large aortic thrombus may detach and cause fatal consequences if not treated timely and effectively, resulting in a large area of intestinal necrosis, renal infarction or lower limb necrosis, and even death. In patients with complex disease, both the mesenteric artery and the renal artery are involved. Surgical thrombectomy may cause massive thrombus shedding, and mesenteric artery or iliac femoral artery embolism. For patients with abdominal aortic thrombosis, a stent was previously used to compress and fix the thrombus in order to reduce the risk of thromboembolism. However, the stent is likely to induce thrombophilia and cause artery occlusion in the long term. Previously, the RSF has been successfully used in our department for the treatment of inferior vena cava thrombosis and has achieved a good clinical curative effect (1,2). In this case, the RSF was used for the first time to treat aortic thrombosis. The RSF, a temporary stent, was able to compress and fix the aortic thrombus, and the long-term complications of stenting, including thrombosis, were avoided after stent removal in this case. This novel technology offers an improved treatment option for patients with thoracic and abdominal aortic thrombosis. Although thrombophilia was the underlying diagnosis as the cause of the contemporary venous and aortic thrombosis in this patient, this diagnosis was not confirmed.

## Conclusions

The RSF was able to compress and fix aortic thrombus without the usual complications of stenting after removal.

## Abbreviations

RSF: Retrieval stent filter
TIPSS: transjugular intrahepatic portosystemic stent shunt
